# Ceramide–Phospholipid Risk Score Associates With Cardiovascular Events and Is Lowered by Atorvastatin, Simvastatin, and Anti-PCSK9 Treatments

**DOI:** 10.1097/FJC.0000000000001817

**Published:** 2026-07-20

**Authors:** Mika Hilvo, Angelika Witoslawska, Mitja Lääperi, Antti Jylhä, Leo-Pekka Lyytikäinen, Hannu Päivä, Jari Laurikka, Winfried März, Pekka J. Karhunen, Terho Lehtimäki, Reijo Laaksonen

**Affiliations:** * VTT Technical Research Centre of Finland Ltd, Espoo, Finland;; † Finnish Cardiovascular Research Center - Tampere, Tampere University, Tampere, Finland;; ‡ Zora Biosciences Oy, Espoo, Finland;; § Faculty of Medicine and Health Technology, Tampere University, and Department of Clinical Chemistry, Fimlab Laboratories. Tampere, Finland;; ¶ Division One, Department of Internal Medicine, Tampere University Hospital, Faculty of Medicine and Health Technology, Tampere University, Tampere, Finland;; ║ Department of Cardio-Thoracic Surgery, Heart Center, Tampere University Hospital; Tampere, Finland;; ** Vth Department of Medicine (Nephrology, Hypertensiology, Endocrinology, Diabetology, Rheumatology, Pneumology), Medical Faculty Mannheim, University of Heidelberg, Mannheim, Germany; and; †† Clinical Institute of Medical and Chemical Laboratory Diagnostics, Medical University of Graz, Graz, Austria.

**Keywords:** ceramide, phospholipid, cardiovascular risk, statin, PCSK9

## Abstract

**Objective:**

The aim of the study was to investigate the prognostic value of previously established ceramide–phospholipid risk score, CERT2, and its response to lipid-lowering treatments in patients with symptomatic or suspected coronary heart disease.

**Approach and results:**

Selected ceramides and phospholipids were analyzed with a quantitative liquid chromatography–mass spectrometry-based method from plasma or serum samples obtained from the Angiography and Genes study (ANGES, N = 966), and placebo-controlled trials investigating atorvastatin and simvastatin (N = 44) and proprotein convertase subtilisin/kexin type 9 (PCSK9) inhibitor RG7652 (N = 78). In the ANGES, the adjusted hazard ratios (95% confidence interval) per SD for the CERT2 risk score were 1.71 (1.42–2.08) for cardiovascular disease death, 1.36 (1.13–1.64) for myocardial infarction, 1.40 (1.20–1.63) for heart failure, 1.23 (1.08–1.40) for atrial fibrillation, 1.09 (0.89–1.33) for stroke, and 1.52 (1.06–2.18) for venous thromboembolism. The median decrease of the CERT2 risk score was 2 points by statin treatment (*P* ≤ 0.001) and 3 points by PCSK9 inhibition (*P* < 0.001) on the 0–12 scale.

**Conclusions:**

The CERT2 risk score is a significant predictor of cardiovascular outcomes in all-comer patients with symptomatic or suspected coronary heart disease. Treatment with lipid-lowering medication significantly lowers the CERT2 risk score.

## Abstract 2

Supplemental Digital Content is Available in the Text.

## INTRODUCTION

Patients with an established coronary heart disease (CHD) with or without a history of myocardial infarction (MI) have a variable risk of cardiovascular (CV) complications such as death, myocardial infarction, or heart failure (HF).^1^ The European Society of Cardiology chronic coronary syndrome 2019 guideline considers >3% annual mortality as a threshold for the very high-risk patients who should receive intensified medical care.^2^ According to this new guideline, risk assessment should be performed for every patient with CHD and it is recommended that the risk stratification should be based on the assessments used to make a diagnosis of CHD. The recommended methods include eg, exercise electrocardiogram, single-photon emission computed tomography or positron emission tomography perfusion, stress echocardiography, cardiac magnetic resonance, coronary computed tomography angiography or invasive coronary angiography (ICA), and invasive functional testing. However, these methods may not be available, or in fact they may not always, eg, ICA alone, provide sufficient information for proper risk assessment. Alternatively, Sabatine et al^3^ have shown that patients with a more recent MI (within the past 2 years), multiple prior MIs, and residual multivessel coronary artery disease (CAD) were at significantly higher risk of cardiovascular disease (CVD) outcomes compared with patients without these risk factors.

In addition to the aforementioned imaging tools and clinical characteristics, validated clinical risk scores can further refine risk stratification in patients with established CHD. The SMART (Second Manifestations of ARTerial disease) risk score estimates the 10-year risk for recurrent CV events in patients with atherosclerotic disease based on easy-to-measure clinical characteristics.^1^ The TIMI (Thrombolysis In Myocardial Infarction) Risk Score for Secondary Prevention, derived from IMPROVE-IT study, similarly predicts long-term recurrent CV events based on 9 clinical characteristics in patients with acute coronary syndrome.^4^ Moreover, actionable biomarkers may also be used in CHD risk assessment. Distinct ceramide and phospholipid molecules have shown to yield an informative risk score (CERT2) in patients with CHD.^5^ This new lipid-based risk biomarker has been shown to associate significantly with the future risk of CVD mortality and events in patients with CHD.^5,6^ Initial reports indicate that serum concentrations of CERT2 components can be lowered with drugs affecting lipid metabolism such as statins, PCSK9 inhibitors, metformin, and fenofibrate.^7–10^ Encouraged by the abovementioned findings, we aimed to validate CERT2 as a prognostic marker in a cohort of all-comers patients with a symptomatic or suspected CHD with a targeted analytical method incorporating labeled isotope standards for each of the CERT2 components. Furthermore, we studied the effect of intensive statin treatment on the CERT2 risk score by using serum samples from an existing trial comparing simvastatin 80 mg/d, atorvastatin 40 mg/d, and placebo, and from a phase II trial that investigated an anti-PCSK9 compound RG7652.

## MATERIALS AND METHODS

### Study Cohorts

Plasma samples were obtained from the Angiography and Genes study (ANGES) study population, which consisted of 966 individuals, who were referred to the Tampere University Hospital (Finland) during years 2002–2004 for coronary angiography because of chest pain and clinically suspected CAD (see **Table**, **Supplemental Digital Content 4**, http://links.lww.com/JCVP/B371). The study has been previously described in more detail.^11^ The median follow-up time was 11.6 years.

Serum samples were obtained from a statin trial that was a randomized, double-blind, placebo-controlled trial with 3 treatment groups: placebo, 40 mg/d atorvastatin, and 80 mg/d simvastatin. A total of 44 subjects (29 men and 15 women) with a median age of 57 years (ranging 50–62) and a diagnosis of hypercholesterolemia were recruited from the University Hospital of Tampere and Primary Health Care Centers of neighboring municipalities of Tampere, Finland, and the duration of the follow-up was 8 weeks. Before the study, the subjects had never been treated with statins. Patients with familial hypercholesterolemia and those with a serum total cholesterol concentration greater than 7.0 mmol/L in the initial screening were excluded, as were women of childbearing potential. More detailed information on the study has been previously published.^12^ The study protocols of the ANGES and the statin trial were accepted by the Ethics Committee of the University Hospital of Tampere, and written informed consent was obtained from all participants.

The PCSK9 trial was a randomized placebo-controlled phase II trial (EQUATOR) for the safe and effective use of RG7652, a fully human monoclonal antibody inhibiting PCSK9 function. In this study (see **Table**, **Supplemental Digital Content 5**, http://links.lww.com/JCVP/B372), fasting plasma samples at baseline and 29 days after treatment were obtained from 80 subjects. The subjects in this subpopulation of the trial were receiving 4 different dose-regimens of RG7652 administration that were equally effective in lowering LDL-C concentration.^13^ The trial (clinicaltrials.gov: NCT01609140) was performed in accordance with the International Conference and Harmonization guidelines and the Declaration of Helsinki, and all patients provided written informed consent by local institutional review boards/ethics committees.

### Prognostic Score

The CERT2 score is a risk assessment tool used to predict the likelihood of CV events. The score consists of 4 components:

- Cer(d18:1/24:1)/Cer(d18:1/24:0),
- Cer(d18:1/16:0)/PC(16:0/22:5),
- Cer(d18:1/18:0)/PC(14:0/22:6),
- PC(16:0/16:0)

The score is calculated by assigning 0–3 points for each of the 4 components for quartiles 1 to 4. These are summed together to give a total score ranging from 0 to 12, which is then stratified into 4 categories representing risk from low to very high (0–3, low; 4–6, moderate; 7–9 high; 10–12 very high) (see **Table**, **Supplemental Digital Content 6**, http://links.lww.com/JCVP/B373). The detailed calculation of the CERT2 risk score has been previously described.*^5^*

### Liquid Chromatography–Tandem Mass Spectrometry (LC-MS/MS) Analysis

Lipids were extracted from serum or plasma samples as previously described.^14^ In short, 10 µL of sample was combined with 590-µL isopropanol:ethyl acetate (8:2), containing the corresponding isotopically labeled standard for each analyte. Samples were then mixed by aspirating them 3 times, followed by 10 minutes centrifugation at 3000 G. Supernatants were transferred to the Eppendorf Twintec PCR plate and sealed with a heat-sealing foil (Hamburg, Germany), before analysis with liquid chromatography–tandem mass spectrometry (LC-MS/MS).

The analyzed lipids and ions used in this study are summarized in **Table**, **Supplemental Digital Content 7**, http://links.lww.com/JCVP/B374. LC-MS/MS analysis was conducted on a Sciex TripleQuad 5500 mass spectrometer coupled to Sciex MPX LC system. This system allows consecutive injections from 2 LCs to be directed into single MS instrument to maximize the sample throughput. Electrospray ionization in positive ion mode was used with multiple reaction monitoring. Instrument and data acquisition were controlled using Analyst (version 1.7). Before the analysis, the method was validated regarding linearity, repeatability, selectivity, specificity, carry-over, sensitivity, accuracy, precision, and dilution. All validation criteria passed the analytical method requirements according to the Biological Method Validation Guidance for Industry (05/24/18). The following settings were applied to all compounds in the analysis: Curtain gas, 35; ion spray voltage, 50000V; temperature, 300°C; gas 1 and gas 2, 50; declustering potential, 30; entrance potential, 10; collision exit potential, 20. Collision energy was set separately for each lipid (see **Table**, **Supplemental Digital Content 7**, http://links.lww.com/JCVP/B375). Chromatographic separation was performed on an Acquity BEH C18 2.1 x 50 mm id.1.7-µm column. Temperature was set to 60°C. Mobile phases consisted of (A) 10 mM of ammonium acetate with 0.1% formic acid and (B) 10 mM of ammonium acetate in acetonitrile:2-propanol (4:3, v/v) with 0.1% formic acid. Loading pump solvent in MPX consisted of A:B (21:79%). The injection volume was 3 µL, and the flow rate was 500 µL/min. The following gradient was applied: A/B (22/78%) from 0 to 1.5 minutes, then B to 85% at 2 minutes and to 100% at 2.5 minutes. B was held at 100% from 2.5 minutes to 4.0 minutes, and then dropped to 78% at 4.1 minutes and held until 4.6 minutes. Both streams had the same parameters. MS analysis was performed from 1.1 minutes to 3.6 minutes which allowed multiplex to run a sample every 2.5 minutes.

### Statistical Methods

Baseline characteristics of the cohorts were described using medians (interquartile ranges) for continuous variables, and numbers (percentages) for categorical variables. The statistical significance between subjects experiencing CVD death versus not was evaluated by Wilcoxon rank-sum test or χ^2^ test, as appropriate. In all analyses, *P* > 0.05 was considered as statistically nonsignificant. Cox proportional hazard regression analyses were used to determine associations with cardiovascular end points. The models were stratified by sex, and in multivariable models, the adjustments were made for age, current smoking, hypertension, BMI, and statin use, and the effects were expressed per SD. Risk curves were constructed with *ggplot2* package using the LOESS method. The quartile cut-offs for CERT2 score calculation^5^ were derived from the ANGES study and applied also to the statin and PCSK9 trials.

## RESULTS

### Baseline Characteristics of the Study Cohorts

Components of the CERT2 risk score, ie, ceramides and phosphatidylcholines, were analyzed in the ANGES study cohort with a quantitative LC-MS/MS method, and the risk score (scale 0–12) was calculated as previously described.^5^ Baseline characteristics of the study subjects are summarized in **Table**, **Supplemental Digital Content 4**, http://links.lww.com/JCVP/B371. Several baseline characteristics were significantly different in patients who died due to CVD reasons during the follow-up compared with patients without fatal CVD events. Patients with fatal events were older, more frequently men, had hypertension, and history of MIs, stroke, or revascularizations. TC, LDL-C, and HDL-C were all in lower concentrations in those patients with future CVD death, whereas triglycerides did not show statistically significant difference between the groups. In this study, all CERT2 components were in significantly higher concentrations in those subjects who experienced CVD death as compared with those who did not.

### CERT2 Risk Score Predicts Cardiovascular Risk

**FIGURE 1.**
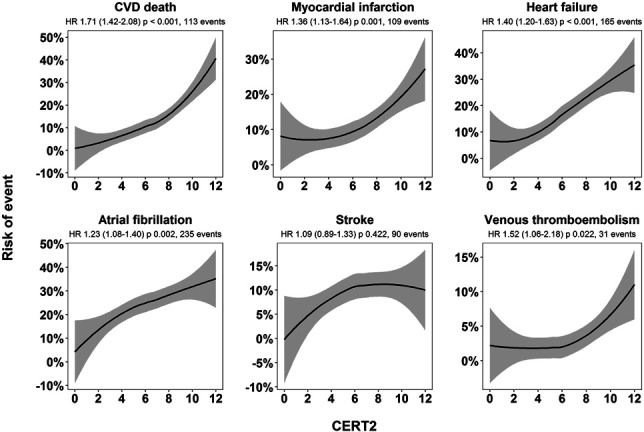
Association between CERT2 score and risk of cardiovascular events. Risk of CVD death, myocardial infarction, heart failure, atrial fibrillation, stroke, and venous thromboembolism across the CERT2 score range (0–12). Hazard ratios (HRs) with 95% confidence intervals, *P*-value, and the number of events are shown in each panel. Higher CERT2 scores were associated with increased risk across most outcomes.

The CERT2 risk score was associated with increased risk of MI, HF, atrial fibrillation (AF), and venous thromboembolism (VTE), whereas a nonsignificant risk association was observed for stroke (Fig. 1). The associations between single CERT2 score components and different CVD outcomes are summarized in the **Table**, **Supplemental Digital Content 8**, http://links.lww.com/JCVP/B375.

### Lipid-Lowering Treatment Decreases CERT2 Risk Score

**FIGURE 2.**
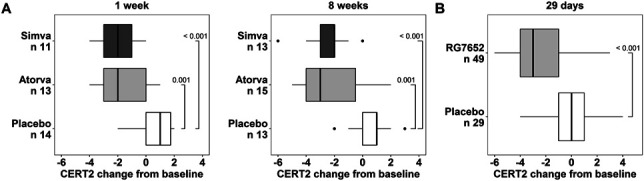
Effect of lipid-lowering interventions on CERT2. Change in CERT2 score from baseline after (A) 1 week and 8 weeks of treatment with simvastatin, atorvastatin, or placebo; (B) 29 days in participants receiving the PCSK9 inhibitor RG7652 or placebo. Both statin therapy and RG7652 significantly reduced CERT2 scores compared with placebo.

Results from the statin trial showed that the median decrease in CERT2 score was 2 points, both after simvastatin (80 mg/day) and atorvastatin (40 mg/day) treatments (Fig. 2A). The decrease was evident already after 1 week since the start of the treatment and remained consistent at the end of the follow-up, ie, 8 weeks. After 1 month of treatment with the PCSK9 inhibitor RG7652, the median decrease of CERT2 score was 3 points (Fig. 2B). The treatment effects of lipid-lowering compounds on the different lipid components of the CERT2 score are shown in **Figures**, **Supplemental Digital Content 1-3**, http://links.lww.com/JCVP/B368, http://links.lww.com/JCVP/B369, http://links.lww.com/JCVP/B370.

## DISCUSSION

The present results validated the prognostic performance of the CERT2 risk score in all-comer patients with symptomatic or suspected CHD. Furthermore, the data showed that intensive statin treatment either with atorvastatin, simvastatin, or an anti-PCSK9 antibody RG7652 significantly lowers the risk score.

Statin treatment resulted in significant reduction in the CERT2 score supporting the initial reports that distinct ceramide species can be lowered by drugs affecting lipid metabolism such as statins and PCSK9 inhibitors.^7,8^ Detailed analyses indicated that both statins and PCSK9 inhibition caused a significant reduction in concentrations of risk-related ceramides Cer(d18:1/16:0), Cer(d18:1/18:0), Cer(18:1/24:1),^6^ and PC(16:0/16:0).^5^ While concentrations of PC species with omega-3 fatty acids remained unchanged particularly during statin treatment, resulting in the CERT2 score lowering, these lipids may carry additional cardiovascular information. In particular, the ratio of eicosatetraenoic (EPA) to arachidonic acid (AA) is a widely recognized marker of coronary heart disease risk. Although CERT2 does not explicitly measure the EPA/AA ratio, several PCs included in the score contain omega-3 fatty acids, such as docosahexaenoic acid. Unlike total circulating EPA, which can fluctuate with diet, supplementation, and other transient factors, omega-3s within PCs are more stable and biologically integrated, potentially providing a more reliable indicator of lipid-mediated risk. By integrating these fatty acid signals with ceramide-mediated pathways, CERT2 captures inflammatory, metabolic, and apoptotic processes beyond the scope of the EPA/AA ratio alone. This suggests that CERT2 may provide similar or even superior risk stratification, highlighting a mechanistic rationale for its strong predictive performance in cardiovascular outcomes.

Our previous analysis of plasma and lipoprotein particles demonstrated that PCSK9 inhibition decreases sphingolipids merely because LDL particles are their major carrier vehicles in the circulation.^8^ Thus, lipid-lowering treatment is an effective method for CERT2 score and ceramide lowering as shown in this study, where the median CERT2 reduction due to statin or PCSK9 treatment was 2–3 points. Although the PCSK9 antibody used in this study is not identical to clinically marketed therapeutic agents, its lipid-lowering potency is comparable, and it is therefore expected that PCSK9 inhibitor achieving similar LDL-cholesterol reductions would exert comparable lowering effects on circulating ceramides and the CERT2 risk score.

In the ANGES study, high-risk subjects (CERT2 score 9 or higher) corresponded to 5%–11% absolute or 27%–38% relative risk reduction for MIs during the 11-year follow-up. Although not an intervention study, these estimates are in line with the observed risk reduction in randomized clinical trials for statins.^15–17^ Thus, the CERT2 risk score could be used to evaluate patient's future CHD risk, the need for different treatments, and gauge the statin treatment efficacy and compliance. However, the assumption that beneficial outcome effects of statin treatment are related to reductions in CERT2 levels needs further data, preferably from large-scale randomized trial with statins. At any rate, identification of high-risk patients with CHD can be useful in clinical decision-making because recent reports have demonstrated greater risk reductions due to PCSK9 inhibitors and ezetimibe in patients at the highest risk compared with those with more moderate risk.^3,4^

In this study, the association between the CERT2 risk score and heart failure was shown in addition to CVD death and MI. In the Cardiovascular Health Study, incident HF has been shown to associate with one of the CERT2 components, Cer(d18:1/16:0).^18^ Furthermore, the Framingham offspring study suggested a potential detrimental impact of higher ceramide ratio Cer(d18:1/16:0)/Cer(d18:1/24:0) on cardiac remodeling traits, which may partly explain the associations of these ceramide species with clinical HF.^19^ By contrast, lower levels of phosphatidylcholines have been recorded in patients with HF in other clinical studies.^20,21^ These previous observations are in line with our results suggesting that particularly distinct ceramide phospholipid ratios are prognostic for incident HF.

Recently, Jensen et al^22^ reported that ceramides and sphingomyelins with palmitic acid were associated with increased atrial fibrillation risk, whereas ceramides and sphingomyelins with very long-chain saturated fatty acids were associated with reduced AF risk. In our study, phosphatidylcholine with palmitic acid (PC16:0/16:0) was associated with incident AF in patients with CHD while individual ceramides showed no association with AF during follow-up. However, a significant association was observed between incident AF and ceramide (d18:1/24:1)/(d18:1/24:0) ratio.

Furthermore, the CERT2 risk score was significantly associated with venous thromboembolism. Earlier, Munzer et al have showed that platelet degranulation and phosphatidylserine exposure fostering thrombin generation and thrombus formation are modified by acid sphingomyelinase-dependent formation of ceramide.^23^ In this study, VTE did not associate with circulating ceramide species, but significant associations were observed between VTE and serum ceramide/phospholipid ratios. The added value of omega-3 phospholipids as prognostic markers is supported by another study that reported that higher levels of n-3 FAs were associated with a lower risk of recurrent VTE or total mortality in elderly patients with VTE.^24^

The limitations of our study include the fact that the statin and PCSK9 trials were small-scale studies. However, the statin trial had multiple sample collections and good compliance to support the validity of the results. The strength of our study was that similar results were obtained with 2 different statins and a PCSK9 inhibitor, suggesting that CERT2 score decrease can be obtained with different kinds of lipid-lowering compounds.

In conclusion, the CERT2 risk score associates significantly with multiple cardiovascular outcomes in all-comers with symptomatic or suspected CHD and high-intensity lipid-lowering treatment lowers CERT2 levels and its components significantly.

## Supplementary Material
