# Supplementary material for: Ceramide–Phospholipid Risk Score Associates With Cardiovascular Events and Is Lowered by Atorvastatin, Simvastatin, and Anti-PCSK9 Treatments
Source: J Cardiovasc Pharmacol. 2026 Jul 20;88(2):161–5. doi: 10.1097/FJC.0000000000001817 (PMC13440561; doi:10.1097/FJC.0000000000001817)
Supplement: Supplementary file 1 [file jcvp-88-161-s001.docx]

**Supplementary Figure S1. Change of CERT2 components after 1 week of simvastatin and atorvastatin treatments.**
