# Supplementary material for: Ceramide–Phospholipid Risk Score Associates With Cardiovascular Events and Is Lowered by Atorvastatin, Simvastatin, and Anti-PCSK9 Treatments
Source: J Cardiovasc Pharmacol. 2026 Jul 20;88(2):161–5. doi: 10.1097/FJC.0000000000001817 (PMC13440561; doi:10.1097/FJC.0000000000001817)
Supplement: Supplementary file 4 [file jcvp-88-161-s004.docx]

**Supplementary Table S1. Demography table of the Angiography and Genes Study cohort.**

| **Variable** | **All samples (N=966)** | **CVD death (N=139)** | **No CVD death (N=827)** | **p-value** |
| --- | --- | --- | --- | --- |
| Age | 63 (55 - 70) | 69 (62 - 74) | 62 (55 - 69) | 9.3E-12 |
| Sex (male) | 633 (64.4%) | 107 (77%) | 514 (62.2%) | 0.001 |
| Current smoker | 136 (13.8%) | 13 (9.4%) | 118 (14.3%) | 0.151 |
| Hypertension | 581 (59.5%) | 96 (69.6%) | 475 (57.8%) | 0.012 |
| Diabetes mellitus | 141 (14.3%) | 45 (32.4%) | 90 (10.9%) | 3.4E-11 |
| Statin treatment | 653 (66.4%) | 84 (60.4%) | 558 (67.5%) | 0.126 |
| BMI | 27.7 (25.0 - 30.5) | 28.4 (25.4 - 31.6) | 27.5 (25.0 - 30.4) | 0.018 |
| History of MI | 240 (24.4%) | 65 (46.8%) | 167 (20.2%) | 2.4E-11 |
| History of CABG | 194 (19.7%) | 39 (28.1%) | 150 (18.1%) | 0.009 |
| History of revascularization | 390 (39.7%) | 68 (48.9%) | 314 (38%) | 0.019 |
| History of stroke | 67 (6.8%) | 25 (18%) | 40 (4.8%) | 3.0E-08 |
| TC (mmol/L) | 4.3 (3.8 - 5.1) | 4.2 (3.6 - 4.7) | 4.4 (3.8 - 5.1) | 0.008 |
| LDL-C (mmol/L) | 2.6 (2.2 - 3.2) | 2.319 (2.0 - 2.8) | 2.7 (2.2 - 3.3) | 0.001 |
| HDL-C (mmol/L) | 1.1 (0.9 - 1.4) | 1.1 (0.8 - 1.3) | 1.2 (0.9 - 1.4) | 0.002 |
| TG (mmol/L) | 1.3 (1.0 - 1.8) | 1.4 (1.1 - 1.8) | 1.3 (1.0 - 1.8) | 0.190 |
| Cer(d18:1/24:1) / Cer(d18:1/24:0) | 0.691 (0.611 - 0.801) | 0.772 (0.657 - 0.869) | 0.684 (0.605 - 0.783) | 1.9E-07 |
| Cer(d18:1/16:0) / PC(16:0/22:5) | 0.010 (0.008 - 0.012) | 0.011 (0.009 - 0.013) | 0.009 (0.008 - 0.011) | 1.2E-07 |
| Cer(d18:1/18:0) / PC(14:0/22:6) | 0.113 (0.077 - 0.163) | 0.134 (0.089 - 0.182) | 0.110 (0.076 - 0.157) | 2.4E-04 |
| PC 16:0/16:0 (µmol/L) | 17.1 (14.4 - 20.2) | 18.3 (15.1 - 21.6) | 16.9 (14.3 - 19.8) | 0.007 |
| *CERT2: 0-3* | 198 (20%) | 12 (8.6%) | 183 (22.2%) | 5.5E-09 |
| *CERT2:4-6* | 361 (36.5%) | 39 (28.1%) | 317 (38.4%) |  |
| *CERT2: 7-8* | 251 (25.4%) | 40 (28.8%) | 204 (24.7%) |  |
| *CERT2: 9-12* | 178 (18%) | 48 (34.5%) | 122 (14.8%) |  |

CVD, cardiovascular disease; BMI, body mass index; MI, myocardial infarction; CABG, coronary artery bypass surgery; TC, total cholesterol; LDL-C; low-density-lipoprotein cholesterol; HDL-C, high-density-lipoprotein cholesterol; TG, triglyceride; Cer, ceramide; PC, phosphatidylcholine; CERT2, coronary event risk test 2.
