# Supplementary material for: Ceramide–Phospholipid Risk Score Associates With Cardiovascular Events and Is Lowered by Atorvastatin, Simvastatin, and Anti-PCSK9 Treatments
Source: J Cardiovasc Pharmacol. 2026 Jul 20;88(2):161–5. doi: 10.1097/FJC.0000000000001817 (PMC13440561; doi:10.1097/FJC.0000000000001817)
Supplement: Supplementary file 5 [file jcvp-88-161-s005.docx]

**Supplementary Table S2. Demography table of the PCSK9 trial substudy.**

| **Variable** | **All samples (N=78)** | **RG7652 (N=49)** | **Placebo (N=29)** |
| --- | --- | --- | --- |
| Age | 65 (58 - 69) | 65 (60 - 68) | 64 (58 - 70) |
| BMI | 29.8 (26.5 - 32.5) | 30.7 (27.1 - 32.6) | 29.8 (26.2 - 31.7) |
| Sex (male) | 40 (51.3%) | 25 (51%) | 15 (51.7%) |
| Diabetes mellitus | 39 (50%) | 25 (51%) | 14 (48.3%) |
| Statin treatment | 53 (67.9%) | 30 (61.2%) | 23 (79.3%) |
| Treatment: 400 mg/4W | 13 (16.7%) | 13 (26.5%) | 0 (0%) |
| Treatment: 400 mg/8W | 12 (15.4%) | 12 (24.5%) | 0 (0%) |
| Treatment: 800 mg/12W | 8 (10.3%) | 8 (16.3%) | 0 (0%) |
| Treatment: 800 mg/8W | 16 (20.5%) | 16 (32.7%) | 0 (0%) |
| Treatment: Placebo | 29 (37.2%) | 0 (0%) | 29 (100%) |

BMI, body mass index.
