# Supplementary material for: Ceramide–Phospholipid Risk Score Associates With Cardiovascular Events and Is Lowered by Atorvastatin, Simvastatin, and Anti-PCSK9 Treatments
Source: J Cardiovasc Pharmacol. 2026 Jul 20;88(2):161–5. doi: 10.1097/FJC.0000000000001817 (PMC13440561; doi:10.1097/FJC.0000000000001817)
Supplement: Supplementary file 6 [file jcvp-88-161-s006.docx]

**Supplementary Table S3****.** **Calculation of the CERT2 score.** CERT2 is composed of one ceramide/ceramide ratio, two ceramide/phosphatidylcholine ratios and one individual phosphatidylcholine species. Each lipid variable was categorized into quartiles (Q1–Q4), and a point score was assigned accordingly. The CERT2 risk score was calculated by summing the point values of each lipid marker. Based on the total CERT2 score (0-12), individuals were stratified into four risk categories.

| **CERT2** | | | **Q1** | | **Q2** | **Q3** | **Q4** | | **Score** |
| --- | --- | --- | --- | --- | --- | --- | --- | --- | --- |
| Cer(d18:1/24:1) / Cer(d18:1/24:0) | | | 0 | | 1 | 2 | 3 | | 0-12 |
| Cer(d18:1/16:0) / PC(16:0/22:5) | | | 0 | | 1 | 2 | 3 | |  |
| Cer(d18:1/18:0) / PC(14:0/22:6) | | | 0 | | 1 | 2 | 3 | |  |
| PC(16:0/16:0) | | | 0 | | 1 | 2 | 3 | |  |
| **Risk group** | | | | | | | | | |
|  | Low | Moderate | | Increased | | | | High | |
| **CERT2** | 0-3 | 4-6 | | 7-8 | | | | 9-12 | |

CERT2, coronary event risk score; Cer, ceramide; PC, phosphatidylcholine; Q, quartile.
