# Supplementary material for: Ceramide–Phospholipid Risk Score Associates With Cardiovascular Events and Is Lowered by Atorvastatin, Simvastatin, and Anti-PCSK9 Treatments
Source: J Cardiovasc Pharmacol. 2026 Jul 20;88(2):161–5. doi: 10.1097/FJC.0000000000001817 (PMC13440561; doi:10.1097/FJC.0000000000001817)
Supplement: Supplementary file 7 [file jcvp-88-161-s007.docx]

**Supplementary Table S4. Lipids and their respective ions used in the study.**

| **Ceramides** | **Q1 (M+HCOO)^-^ (m/z)** | **Q1 (M+HCOO)^-^ (m/z)** | **Collision energy** | **Internal standard** |
| --- | --- | --- | --- | --- |
| 18:1/16:0 | 538.5 | 264.25 | 40 | 18:1/16:0 – d4 |
| 18:1/18:0 | 566.5 | 264.25 | 40 | 18:1/18:0 – d4 |
| 18:1/24:0 | 650.6 | 264.25 | 40 | 18:1/24:0 – d4 |
| 18:1/24:1 | 648.6 | 264.25 | 40 | 18:1/24:1 – d4 |
| **Phosphatidylcholines** |  |  |  |  |
| 14:0/22:6 | 778.5 | 184.1 | 40 | 14:0/22:6 – d9 |
| 16:0/16:0 | 734.6 | 184.1 | 15 | 16:0/16:0 – d9 |
| 16:0/22:5 | 808.6 | 184.1 | 15 | 16:0/22:5 – d9 |
