# Supplementary material for: Ceramide–Phospholipid Risk Score Associates With Cardiovascular Events and Is Lowered by Atorvastatin, Simvastatin, and Anti-PCSK9 Treatments
Source: J Cardiovasc Pharmacol. 2026 Jul 20;88(2):161–5. doi: 10.1097/FJC.0000000000001817 (PMC13440561; doi:10.1097/FJC.0000000000001817)
Supplement: Supplementary file 8 [file jcvp-88-161-s008.docx]

**Supplementary Table S5. HRs for CERT2 components predicting cardiovascular events.** HRs are shown per SD of lipid or lipid ratio change, and the models were adjusted for age, current smoking, hypertension, BMI, statin use and stratified by sex.

|  | **CVD death** | | **MI** | | **Heart failure** | |
| --- | --- | --- | --- | --- | --- | --- |
| **Variable** | **HR (95% CI)** | **p-value** | **HR (95% CI)** | **p-value** | **HR (95% CI)** | **p-value** |
| Cer(d18:1/16:0) | 1.31 (1.09-1.58) | **0.005** | 1.24 (1.03-1.50) | **0.026** | 1.12 (0.95-1.31) | 0.186 |
| Cer(d18:1/18:0) | 0.96 (0.80-1.16) | 0.685 | 1.08 (0.90-1.29) | 0.417 | 1.04 (0.89-1.21) | 0.625 |
| Cer(d18:1/24:0) | 0.86 (0.72-1.03) | 0.096 | 0.97 (0.81-1.16) | 0.738 | 0.86 (0.74-1.00) | **0.049** |
| Cer(d18:1/24:1) | 1.20 (1.00-1.45) | 0.055 | 1.17 (0.97-1.41) | 0.105 | 1.12 (0.95-1.31) | 0.179 |
| PC(14:0/22:6) | 0.63 (0.53-0.76) | **1.6E-06** | 0.76 (0.63-0.92) | **0.004** | 0.81 (0.69-0.96) | **0.013** |
| PC(16:0/22:5) | 0.86 (0.73-1.03) | 0.103 | 0.86 (0.73-1.03) | 0.095 | 0.87 (0.75-1.01) | 0.060 |
| PC(16:0/16:0) | 1.31 (1.08-1.60) | **0.006** | 1.03 (0.85-1.24) | 0.782 | 1.18 (1.00-1.40) | **0.050** |
| Cer(d18:1/24:1) / Cer(d18:1/24:0) | 1.53 (1.27-1.84) | **6.5E-06** | 1.25 (1.04-1.51) | **0.019** | 1.41 (1.20-1.65) | **2.4E-05** |
| Cer(d18:1/16:0) / PC(16:0/22:5) | 1.43 (1.19-1.71) | **9.8E-05** | 1.38 (1.15-1.66) | **4.4E-04** | 1.26 (1.08-1.46) | **0.003** |
| Cer(d18:1/18:0) / PC(14:0/22:6) | 1.41 (1.17-1.70) | **3.5E-04** | 1.30 (1.08-1.56) | **0.006** | 1.20 (1.02-1.41) | **0.026** |
|  | **Atrial fibrillation** | | **Stroke** | | **Venous thromboembolism** | |
| **Variable** | **HR (95% CI)** | **p-value** | **HR (95% CI)** | **p-value** | **HR (95% CI)** | **p-value** |
| Cer(d18:1/16:0) | 1.03 (0.90-1.18) | 0.654 | 1.03 (0.83-1.28) | 0.780 | 1.07 (0.76-1.52) | 0.692 |
| Cer(d18:1/18:0) | 1.05 (0.93-1.20) | 0.431 | 1.01 (0.82-1.25) | 0.909 | 1.18 (0.84-1.65) | 0.348 |
| Cer(d18:1/24:0) | 0.91 (0.80-1.03) | 0.143 | 0.96 (0.77-1.19) | 0.698 | 0.79 (0.57-1.09) | 0.148 |
| Cer(d18:1/24:1) | 1.07 (0.94-1.22) | 0.325 | 1.01 (0.82-1.25) | 0.911 | 0.97 (0.69-1.37) | 0.865 |
| PC(14:0/22:6) | 0.89 (0.77-1.01) | 0.075 | 0.89 (0.72-1.11) | 0.313 | 0.64 (0.46-0.89) | **0.007** |
| PC(16:0/22:5) | 1.05 (0.92-1.19) | 0.464 | 1.07 (0.87-1.32) | 0.535 | 0.67 (0.51-0.88) | **0.004** |
| PC(16:0/16:0) | 1.17 (1.02-1.34) | **0.023** | 1.03 (0.83-1.29) | 0.769 | 0.96 (0.68-1.35) | 0.809 |
| Cer(d18:1/24:1) / Cer(d18:1/24:0) | 1.23 (1.08-1.40) | **0.002** | 1.07 (0.87-1.33) | 0.530 | 1.36 (0.95-1.95) | 0.089 |
| Cer(d18:1/16:0) / PC(16:0/22:5) | 0.98 (0.86-1.11) | 0.727 | 0.96 (0.78-1.19) | 0.714 | 1.73 (1.24-2.41) | **0.001** |
| Cer(d18:1/18:0) / PC(14:0/22:6) | 1.14 (1.00-1.30) | 0.058 | 1.10 (0.89-1.36) | 0.394 | 1.58 (1.13-2.21) | **0.007** |

CVD, cardiovascular disease; MI, myocardial infarction; Cer, ceramide; PC, phosphatidylcholine; HR, hazard ratio; CI, confidence interval.
